# Estrogen Receptor-A in Medial Preoptic Area Contributes to Sex Difference of Mice in Response to Sevoflurane Anesthesia

**DOI:** 10.1007/s12264-022-00825-w

**Published:** 2022-02-17

**Authors:** Yunyun Zhang, Huiming Li, Xinxin Zhang, Sa Wang, Dan Wang, Jiajia Wang, Tingting Tong, Zhen Zhang, Qianzi Yang, Hailong Dong

**Affiliations:** Department of Anesthesiology and Perioperative Medicine, Xijing Hospital, The Fourth Military Medical University, Xi’an, 710032 China

**Keywords:** Sex difference, Anesthesia, Estrogen receptor alpha, Medial preoptic area, Sevoflurane

## Abstract

A growing number of studies have identified sex differences in response to general anesthesia; however, the underlying neural mechanisms are unclear. The medial preoptic area (MPA), an important sexually dimorphic structure and a critical hub for regulating consciousness transition, is enriched with estrogen receptor alpha (ERα), particularly in neuronal clusters that participate in regulating sleep. We found that male mice were more sensitive to sevoflurane. Pharmacological inhibition of ERα in the MPA abolished the sex differences in sevoflurane anesthesia, in particular by extending the induction time and facilitating emergence in males but not in females. Suppression of ERα *in vitro* inhibited GABAergic and glutamatergic neurons of the MPA in males but not in females. Furthermore, ERα knockdown in GABAergic neurons of the male MPA was sufficient to eliminate sex differences during sevoflurane anesthesia. Collectively, MPA ERα positively regulates the activity of MPA GABAergic neurons in males but not in females, which contributes to the sex difference of mice in sevoflurane anesthesia.

**Supplementary Information:**

The online version contains supplementary material available at 10.1007/s12264-022-00825-w.

## Introduction

General anesthesia, with the features of unconsciousness, analgesia, amnesia, and immobility, has been widely used in surgery for more than 170 years. However, the underlying mechanisms remain largely unknown. Accumulating evidence has shown that unconsciousness induced by general anesthetics shares the neural pathway with sleep [1], and many sleep-related nuclei and networks have been reported to be involved in anesthesia [2–4]. However, in both basic and clinical research, females have been primarily excluded for a long time because of the confounding variables of breeding, pregnancy, and hormonal fluctuations that are characteristic of this sex. Considering that half of the surgical population is female, the exclusion of this sex from anesthesia-related studies has produced a huge knowledge gap from the literature to the clinic.

In the past decade, a small but growing number of investigations have been focused on sex differences in response to general anesthesia [5–13]. Some clinical studies have demonstrated that women require a higher propofol infusion rate to maintain general anesthesia [5] and spend less time emerging from anesthesia than men do [6–8]. However, Kodaka *et al*. [9] reported that there was no difference in the minimum alveolar anesthetic concentration (MAC) requirement or bispectral index (BIS) value at loss of consciousness between men and women for sevoflurane anesthesia. A few basic studies with rodent models have reported that male rats require larger doses of anesthetics to produce general anesthesia [10, 11] and emerge faster from a single intraperitoneal injection of propofol than female rats [12]. The discrepancy in previous studies has not yet provided a clear view of sex differences in general anesthesia. Nevertheless, the traditional explanations for the previously reported sex differences in clinical anesthetic effects involve sex-specific pharmacokinetic disparities [14–20] and hormonal differences [10, 21–28]. Whether gender-related neural mechanisms contribute to sex-specific responses to general anesthetics remains unclear.

The preoptic area (POA), one of the most celebrated sexually dimorphic structures [29–31], is enriched with estrogen receptor alpha (ERα, gene *ESR1*) [32, 33]. ERα expression in the POA has a sex preference, and its expression is higher in females than in males [34]. The POA has also been identified as a critical hub for sleep generation. Inhibitory neurons in the POA, such as GABAergic [35] neurons, have sleep-promoting and sleep-active features and project inhibitory innervation to the arousal-promoting systems [36, 37]. In contrast, excitatory glutamatergic neurons in the POA promote wakefulness [38]. Single-cell RNA sequencing of the POA has revealed that *ESR1* is densely co-expressed in neuronal clusters that participate in regulating sleep-wakefulness [33]. The anesthesia-regulatory role of POA has been identified in the presence of dexmedetomidine [39], propofol [40, 41], and volatile anesthetics [4]. Therefore, the POA offers a potential target for coding sex differences in general anesthesia.

In the current study, we aimed to examine sex-specific differences in the murine response to sevoflurane. Considering that ERα is expressed most densely in the medial POA (MPA) in both male and female mice [32, 33], the pharmacological technique, patch-clamp recordings, and short-hairpin RNA knockdown were applied to further investigate the role of MPA ERα in sevoflurane anesthesia in both sexes in an attempt to partially explain the sex differences in the response to sevoflurane anesthesia.

## Materials and Methods

### Animals

C57BL/6J mice used in this study were purchased from the Beijing Vital River Laboratory Animal Technology Co., Ltd. Vglut_2_-ires-Cre (Stock No. 028863; Jackson Laboratories, Bar Harbor, ME, USA) or Vgat-ires-Cre mice (Stock No. 028862; Jackson Laboratories, Bar Harbor, ME, USA) were crossed with Cre-dependent tdTomato reporter knock-in mice (B-tdTomato cKI mice, provided by Beijing Biocytogen Inc.) to generate mice expressing red fluorescence in the major subset of glutamatergic (Vglut2-Tdtomato mice) or GABAergic (Vgat-tdTomato mice) neurons. Mice were housed at 18–23°C with 38%–42% humidity in a 12-h light–dark cycle (lights on 07:00–19:00) with free access to food and water. All experiments were performed on age-matched (8–12 weeks old) adult male (24–27 g) and female (22–25 g) mice during the light cycle (9:00–18:00). All studies were carried out in accordance with the protocols approved by the Animal Experiment Ethics Committee and strictly in compliance with the guidelines for animal experiments of the Fourth Military Medical University (Xi’an, China) as well as the ARRIVE guidelines.

### Short-hairpin RNA and Virus Preparation

ERα short-hairpin RNA sequences were designed based on the report by Musatov *et al*. [42, 43]. ERα–short-hairpin RNA (shERα, *ESR1*, 5′-GGCATGGAGCATCTCTACA-3′), or short-hairpin control (a scrambled sequence) was ligated into the designated plasmid vector construct (pAAV–GAD67–EGFP–3xFLAG-WPRE), which was designed to co-express the short-hairpin RNA driven by the GAD67 promoter and the enhanced green fluorescent protein. The reconstructed vector was packaged in adeno-associated virus 2/9 (AAV2/9) serotype with titers of 1.0 × 10^12^ genome copies/mL. The reconstruction and packaging processes were completed by Obio Technology Corp., Ltd. (Shanghai, China).

### Stereotaxic surgery

Mice were fixed in a stereotaxic frame (RWD, Shenzhen, China) under 1.4–1.5 vol% isoflurane anesthesia with erythromycin ophthalmic ointment applied for eye protection. After shaving and skin antisepsis, 1% lidocaine was subcutaneously injected and a sagittal incision was made in the scalp. During surgery, the mice were kept warm using a heating plate.

For pharmacological experiments, a custom-made double guide cannula (center-to-center distance 0.8 mm, 0.48 mm in diameter, RWD, Shenzhen, China) was inserted bilaterally into the MPA (AP: + 0.15 mm; ML: ± 0.4 mm; DV: − 4.5 mm from the brain surface) of C57BL/6J mice. A double dummy cannula (RWD, Shenzhen, China) secured with a dust cap was inserted into the guide cannula to prevent clogging during the recovery period.

For the ERα knockdown test, AAV9–GAD67–EGFP–shERα-WPRE or AAV9–GAD67–EGFP–short-hairpin control was bilaterally injected (300 nL/side, 50 nL/min) into the MPA (AP: + 0.15 mm; ML: ± 0.4 mm; DV: − 5.0 mm from the brain surface). After microinjection, the micropipette was left in place for an additional 10 min to minimize the spread of the virus along the injection track.

After cannula insertion and virus injection, three stainless steel screws (1 × 5 mm^2^, RWD, Shenzhen, China) were secured on the skull surface as electroencephalogram (EEG) electrodes: the positive electrode on one side of the frontal cortex (AP: − 1.5 mm, ML: + 1.5 mm), the negative electrode on the other side of the parietal cortex (AP: + 1.5 mm, ML: −1.5 mm), and a reference electrode at the occipital cortex (AP: − 5.5 mm, ML: 0.0 mm). The cannula and skull screws were fixed using methyl methacrylate cement. After surgery, the mice were moved to a heating plate until they recovered consciousness. Meloxicam (0.03 mg/kg) was used for postoperative analgesia for 3 days.

### Measurement of Induction and Emergence Times

After habituation in a horizontal Plexiglas^®^ cylinder (45 cm long, 12 cm in diameter) for 2 h for 3 consecutive days, the duration of sevoflurane induction or emergence was tested using the cylinder, in which mice were administered 2.4 vol% sevoflurane in 1 L/min 100% oxygen using a sevoflurane vaporizer (RWD, Shenzhen, China). The inhaled concentrations of sevoflurane were continuously monitored using a gas analyzer (G60; Philips, Shenzhen, China) with sampling from the outlet of the cylinder. The cylinder was rotated 90° every 15 s after the sevoflurane inhalation was started or stopped, and loss of the righting reflex (LORR) was defined if mice could not turn prone onto four limbs and remained in the supine position for >60 s. Induction time was defined as the duration from the onset of anesthetic inhalation to LORR. After LORR, rotation of the cylinder was stopped, and 2.4 vol% sevoflurane was continuously administered for an additional 30 min to ensure equilibration. Mice were considered to have achieved recovery of the righting reflex (RORR) if they could turn themselves to the prone position. Emergence time was defined as the interval from the cessation of anesthetic inhalation to RORR. For the cannula microinjection experiment, considering the effective time of 1,3-bis (4-hydroxyphenyl)-4-methyl-5-[4-(2-piperidinylethoxy) phenol]-1H-pyrazole dihydrochloride (MPP), induction time and emergence time were assessed in two separate trials (Fig. 2B, C).

### Pharmacological Experiments

The ERα antagonist MPP was from Tocris Bioscience (Catalog number: 1991, United Kingdom). MPP was dissolved in dimethyl sulfoxide (DMSO; Sigma, Billerica, MA, USA) as a stock solution (1 mg/mL) and diluted to 1 ng/μL with artificial cerebrospinal fluid (ACSF) for the formal experiment (DMSO = 0.1%).

After mice had recovered from surgery for at least 5–7 days, MPP (1 ng/μL, 0.3 μL/side) was microinjected through the double injector cannula, which had a 0.5-mm extension beyond the tip of the guide cannula. Once the injection was completed, the injector cannula was left in place for an additional 5 min to prevent spread of the drug along the injection track.

### EEG Recording and Analysis

EEG signals were continuously recorded using the Power Lab 16/35 amplifier system (PL3516, AD Instruments, New Zealand) and LabChart Pro V8.1.13 software (MLU60/8, AD Instruments). Raw EEG data were digitized at 1000 Hz.

To calculate the burst-suppression ratio (BSR, a marker of deep anesthesia), EEG data were bandpass filtered at 5–30 Hz, and then analyzed using a custom Matlab script (R2019a, MathWorks, Natick, MA, USA). A suppression event, with an assignment of 1, was defined as when the EEG amplitude was less than its individual threshold for >0.5 s. Otherwise, the amplitude above the threshold was defined as a burst event assigned a value of 0. The BSR was calculated every 1 min by the percentage of suppression events as required. The presence of a BSR was defined as a certain minute when the BSR was >20%, and the extinction of BSR was defined as the time interval from the cessation of anesthetic administration to the end of the last suppression event.

For spectral analysis, the spectrum of frequencies throughout the entire procedure within the 0.3–50 Hz range was plotted for all mice. The relative power in the delta band (1–4 Hz), computed by averaging the signal power across the frequency range of the delta band and then dividing by the total power, was recorded for 5 min after the onset and cessation of sevoflurane administration.

### Slice Preparation and Whole-Cell Recordings

Acute coronal brain slices containing the MPA at 300 µm thickness were cut on a vibratome in ice-cold cutting solution (in mmol/L): 92 NMDG, 2.5 KCl, 1.25 NaH_2_PO_4_, 30 NaHCO_3_, 20 HEPES, 25 glucose, 2 thiourea, 5 Na-ascorbate, 3 Na-pyruvate, 0.5 CaCl_2_, and 10 MgSO_4_ (bubbled with 95%O_2_ and 5%CO_2_). The slices were immersed in ACSF (in mmol/L): 124 NaCl, 2.5 KCl, 1.25 NaH_2_PO_4_, 24 NaHCO_3_, 12.5 glucose, 5 HEPES, 2 CaCl_2_, and 2 MgSO_4_ for 45 min at 35°C and then 1 h at room temperature (22°C–26°C) before being transferred to the recording chamber. Patch pipettes (3 μm–5 μm, 4 MΩ–6 MΩ) for whole-cell recording were filled with an internal solution containing (in mmol/L) 145 K-gluconate, 10 HEPES, 1 EGTA, 2 Mg-ATP, 0.3 Na_2_-GTP, and 2 MgCl_2_. Bright tdTomato-fluorescence or EGFP-fluorescence of glutamatergic or GABAergic neurons in the MPA were visually identified using an Olympus U-HGLGPS fluorescent source microscope. Whole-cell recordings were made while perfusing (3–5 mL/min) ACSF containing estradiol (10 nmol/L) at room temperature. Most of the MPA neurons exhibited spontaneous action potential (AP) firing; for these neurons, we waited for 5 min–10 min after the establishment of whole-cell patching configuration to record stable responses. In neurons without spontaneous firing, a positive current (10 pA–50 pA) was injected *via* the recording pipette to bring the resting membrane potential to 45 mV–50 mV and then to induce steady firing activity [44, 45]. MPP (1 μmol/L or 5 μmol/L, MPP was dissolved into DMSO as a stock solution (5 mmol/L), and diluted to 5 μmol/L (DMSO = 0.1%) or 1 μmol/L (DMSO = 0.02%) with ACSF for the formal experiment) was bath applied for 15 min after 5 min of baseline recording. We averaged the number of APs recorded during the 5 min baseline and the last 5 min of MPP application for comparison. All tested neurons were checked for the co-expression of ERα after patching, and only data from ERα-positive neurons were analyzed. Data were acquired using pClamp 10.6 (Molecular Devices, Foster City, CA, USA). AP numbers were calculated using Mini Analysis 6.0.1 (Synaptosoft Inc., Decatur, GA, USA).

### Histological Verification

Mice were deeply anesthetized with isoflurane and perfused with 4% paraformaldehyde (PFA) followed by 0.9% saline [46]. Brains were post-fixed for 2 h in 4% PFA at 4°C and then dehydrated in 30% sucrose in phosphate-buffered saline (PBS) at 4°C until sinking. Brains were coronally cut at 40 μm on a cryostat microtome (CM1200 Leica, Wetzlar, Germany). The sections were washed three times for 10 min each in PBS and then blocked with 5% normal donkey serum in PBS with 0.3% Triton X-100 for 2 h at room temperature. The primary antibodies, rabbit anti–ERα (1:200, Abcam, ab32063, United Kingdom), were incubated at 4°C for 48 h, followed by incubation with donkey anti-rabbit Alexa Fluor 594 (1:500, Jackson ImmunoResearch, Code: 711-585-152, West Grove, PA, USA) as secondary antibodies for 2 h at room temperature. After another 3 × 10 min wash with PBS, the sections were mounted in Fluoromount-G (Millipore Sigma, Billerica, MA, USA), and images of immunostaining were captured using a laser confocal microscope (FV1200, Olympus, Tokyo, Japan).

### Western Blotting

Mice were deeply anesthetized with isoflurane before transcardial infusion of 0.9% saline, and their brains were quickly removed. The MPA was dissected on ice using a mouse brain matrix (RWD, Shenzhen, China). Total proteins were isolated using RIPA lysis buffer supplemented with a protease inhibitor cocktail (catalog number: 78438, Thermo Fisher Scientific, Carlsbad, CA, USA). Equal amounts of protein (~50 μg) were loaded and separated on 10% Tris–Tricine SDS-PAGE gels and transferred to polyvinylidene difluoride membranes. The membranes were blocked in 5% nonfat milk for 1 h and incubated overnight with primary antibodies at 4°C. After incubation with secondary antibodies (1:5000, Zhuangzhi Biological Technology Co., Ltd, EK020/EK010, Xi'an, China) for 1 h at room temperature, the signals were visualized using enhanced chemiluminescence (Millipore Sigma, Billerica, MA, USA) and captured using a ChemiDocXRS system (Bio-Rad, Minnesota, USA). Western blot results were quantified using densitometry (Image Lab). The primary antibodies used were rabbit anti–ERα (1:1000, Abcam, ab32063, United Kingdom) and a glyceraldehyde-3-phosphate dehydrogenase antibody (1:10000, Zhuangzhi Biological Technology Co., Ltd, NC020, Xi'an, China).

### Measurement of Body Surface Temperature

The back skin surface temperature of males and females (*n* = 6) was measured before and 10 min, 20 min, 30 min, 40 min, 50 min, and 60 min after injection of MPP to determine the temperature response to MPP in male and female mice. An infrared thermometer (YHW-2, Yuwell, Jiangsu, China) was used as previously described [47].

### Statistical Analysis

Prism 8.0 (GraphPad Software, San Diego, CA, USA) was used for statistical analysis. Data are presented as the mean ± standard error of the mean or the mean ± standard deviation as needed. All sets of data were verified for normality and homogeneity of variance using Kolmogorov–Smirnov and Brown–Forsythe tests before analysis. Student’s *t*-test was used to assess the statistical significance for comparisons of two groups, followed by two-way analysis of variance (ANOVA) followed by Bonferroni’s multiple comparisons for three or more groups. Statistical significance was set at *P* < 0.05.

## Results

### Sevoflurane Induces Sex Differences Across Anesthesia Induction, Maintenance, and Emergence

**Fig. 1:**
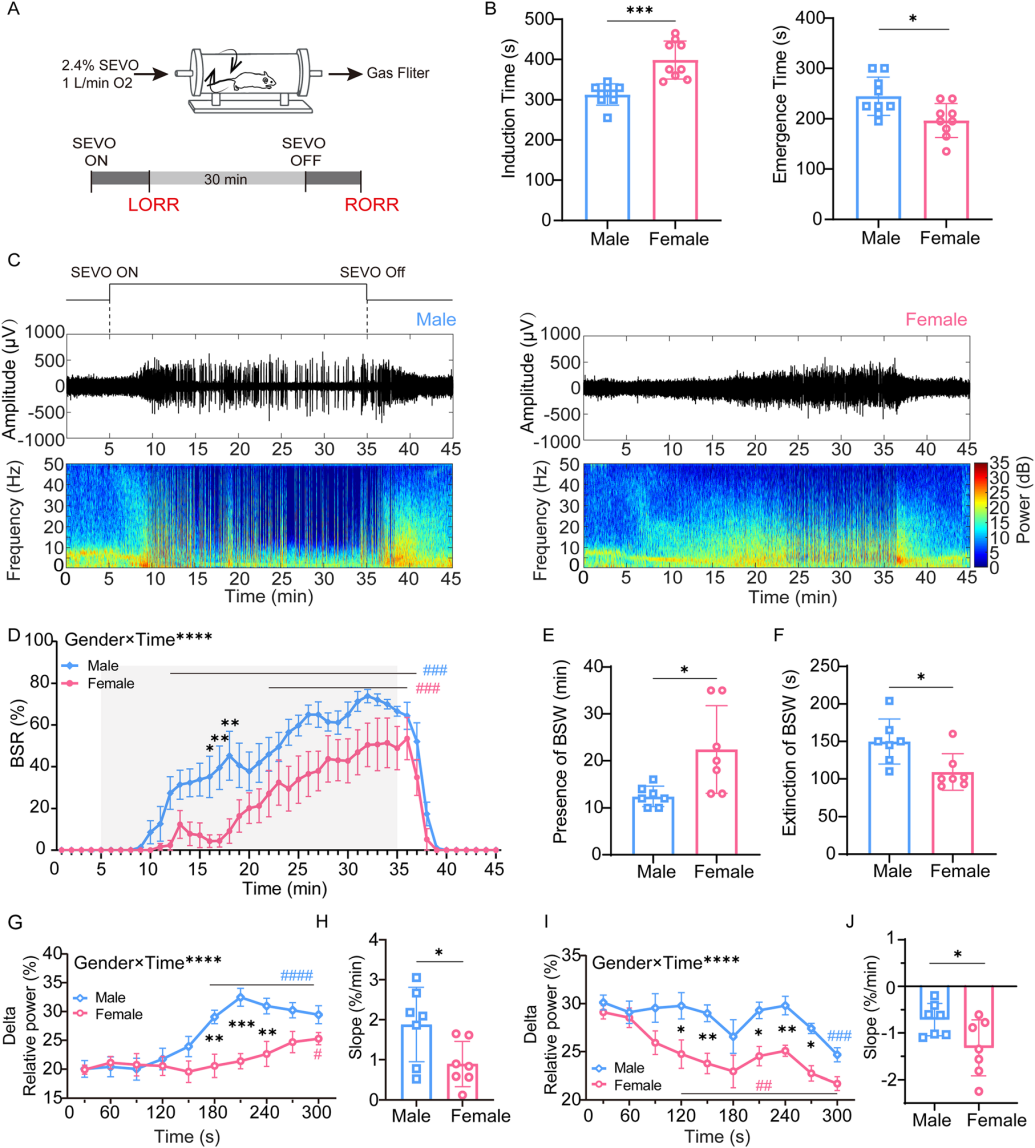
Sex-specific differences in behavioral response and electroencephalogram pattern in mice during sevoflurane anesthesia. **A** Schematic of Plexiglas cylinder and protocol used in behavioral observations. **B** Induction time (left) and emergence time (right) in male and female mice (*t*_16_ = 4.772, *P* = 0.0002 for induction time; *t*_16_ =2.837, *P* = 0.0119 for emergence time; two-tailed unpaired Student’s *t*-test). **C** Representative electroencephalogram traces and corresponding power spectrograms in a male and a female recorded throughout the process of sevoflurane anesthesia.** D** BSR throughout the electroencephalogram recording (45 min) in males and females. BSR is calculated every minute (*F*_44,528_ = 2.341, *P* < 0.0001; two-way analysis of variance (ANOVA) followed by *post hoc* Bonferroni’s multiple comparisons). **E, F** Presence (**E**) and extinction (**F**) of BSR in male and female groups (*t*_12_ = 2.770, *P* = 0.0170 for presence of BSR, *t*_12_ = 2.781, *P* = 0.0166 for extinction of BSR; two-tailed unpaired Student’s *t*-test). **G, I** Spectral analysis of relative power in the delta band between male and female groups during induction (**G**, 5 min after administration of sevoflurane) and emergence (**I**, 5 min after cessation of administration). The relative delta power is computed every 30 s (*F*_9,108_ = 4.997, *P* < 0.0001 for induction; *F*_9, 108_ = 1.987, *P* = 0.0476 for emergence; two-way ANOVA followed by *post hoc* Bonferroni’s multiple comparisons). **H, J** Relative power transition rate in delta wave between male and female groups during induction (**H**) and emergence (**J**) (*t*_12_ = 2.395, *P* = 0.0338 for induction; *t*_12_ = 2.320, *P* = 0.0387 for emergence; two-tailed unpaired Student’s *t*-test; *significant difference between males and females; ^#^significant difference from initial value). SEVO, sevoflurane; BSR, burst suppression ratio; BSW, burst suppression wave; LORR, loss of righting reflex; RORR, recovery of righting reflex.

To determine whether there was a sex difference in the anesthetic effect of sevoflurane in mice, age-matched adult female and male mice were used to compare the time spent in losing and regaining consciousness (in terms of the righting reflex) from sevoflurane anesthesia (Fig. 1A). We found that female mice took longer to lose the righting reflex than male mice (398.9 ± 46.8 s *vs* 313.3 ± 26.5 s, *P* = 0.0002, *n* = 9 per group; Fig. 1B), and less time to emerge from anesthesia after sevoflurane exposure ceased (196.7 ± 33.9 s *vs* 245 ± 38.2 s, *P* = 0.0119, *n* = 9 per group; Fig. 1B).

To investigate the sex difference in response to sevoflurane in EEG patterns during anesthesia maintenance, we continuously recorded EEG signals over the peri-anesthesia period (Fig. 1C). Continuous inhalation of 2.4 vol% sevoflurane for 30 min after LORR kept animals anesthetized. However, female mice experienced fewer burst suppression patterns than male mice, suggesting lighter anesthesia. Although the BSR increased in both sexes as anesthesia continued, females displayed a rather lower BSR than males (*F*_44, 528_ = 2.341, *P* < 0.0001, *n* = 7 per group; Fig. 1D). Moreover, the presence of burst suppression patterns was later in females than males (22.4 ± 9.3 min *vs* 12.4 ± 2.1 min, *P* = 0.017, *n* = 7 per group; Fig. 1E). In addition, the extinction of burst suppression patterns in females was faster than in males (109.3 ± 24.3 s *vs* 149.9 ± 30.0 s, *P* = 0.0116, *n* = 7 per group; Fig. 1F). The enhancement of delta oscillation also represents the deepening of anesthesia; therefore, we analyzed the relative power in the delta band every 30 s during induction and emergence. Delta power gradually increased in both males and females from the start of sevoflurane inhalation, but males had a stronger delta oscillation than females (Fig. 1G). Also, delta power increased faster in males than females during induction (1.9 ± 0.9%/min *vs* 0.9 ± 0.6%/min, *P* = 0.0338, *n* = 7 per group; Fig. 1H). During emergence, delta power gradually decreased in both sexes, but males maintained a higher power than females (Fig. 1I), and the rate of decrease in power was significantly slower in males than in females (–0.7 ± 0.3%/min *vs* –1.3 ± 0.6%/min, *P* = 0.0387, *n* = 7 per group; Fig. 1J). The above results suggest that sevoflurane anesthesia has a sex-dependent effect in mice, and males are more sensitive to sevoflurane than females.

### Inhibition of ERα in the MPA Alters Anesthesia Sensitivity and Depth in Male Mice

**Fig. 2:**
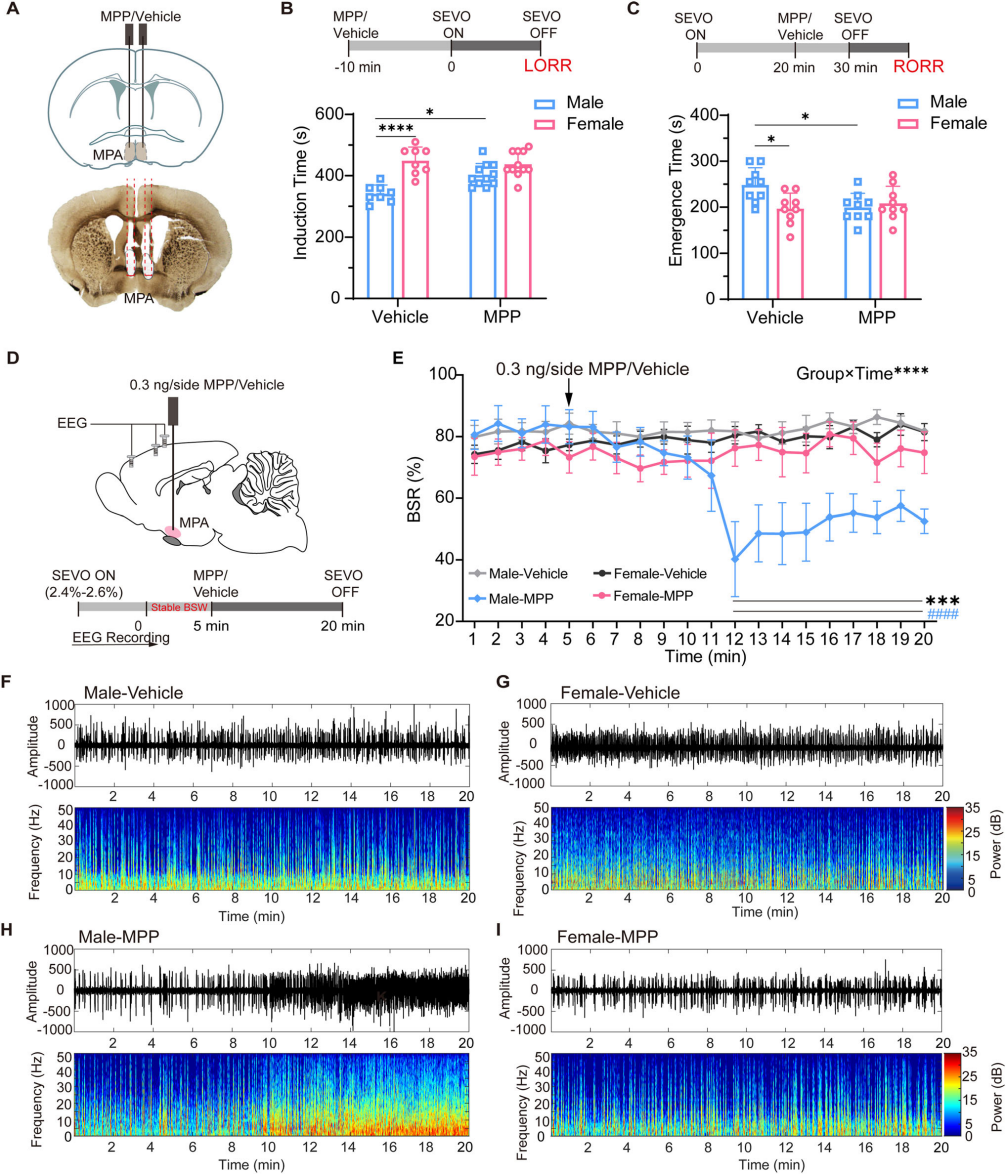
Inhibition of ERα in the MPA reduces the anesthesia sensitivity and anesthesia depth in male mice. **A** Diagram of coronal brain section (upper) showing cannula insertion and histological image (lower) showing the bilateral location of cannulas in the MPA. **B, C** Schematics of protocols (upper) and results (lower) for induction (**B**) and emergence (**C**) times in male and female mice in the vehicle and MPP groups (*F*_1,34_ = 8.144, *P* = 0.0073; *t*_34_ = 5.587 (Vehicle: male *vs* Vehicle: female), *P* < 0.0001; *t*_34_ = 3.401 (Vehicle: male *vs* MPP: male), *P* = 0.0104 for induction time (**B**); *F*_1,32_ = 6.757, *P* = 0.0140; *t*_32_ = 3.137 (Vehicle: male *vs* Vehicle: female), *P* = 0.0219; *t*_32_ = 2.968 (Vehicle: male *vs* MPP: male), *P* = 0.0338 for emergence time (**C**); two-way analysis of variance (ANOVA) followed by *post hoc* Bonferroni’s multiple comparisons). **D** Diagram of sagittal brain section showing the site of cannula implantation and schematic protocol for EEG recording. **E** BSR at 5 min before and 15 min after MPP/vehicle injection into the MPA in male and female mice. BSR is computed each minute (male-vehicle: *n* = 5; female-vehicle: *n* = 5; male-MPP: *n* = 6; female-MPP: *n* = 5; *F*_57,323_ = 6.426, *P* < 0.0001; two-way ANOVA followed by *post hoc* Bonferroni’s multiple comparisons; *significant difference between male-vehicle and male-MPP groups; ^#^significant difference from initial value). **F–I** Representative EEG traces and power spectrograms over time after MPP (lower) or vehicle (upper) injection in male (left) and female (right) mice. MPA, medial preoptic area; SEVO, sevoflurane; BSW, burst suppression wave; BSR, burst suppression ratio; LORR, loss of righting reflex; RORR, recovery of righting reflex; EEG, electroencephalogram.

To determine the regulatory role of MPA ERα in the sevoflurane anesthesia-induced sex difference, MPP, a highly selective ERα antagonist, was bilaterally microinjected into the MPA of male and female mice (Fig. 2A). MPP was administered 10 min before starting or stopping sevoflurane inhalation to assess the changes in LORR or RORR, since MPP requires 5 min–10 min to take effect (Fig. 2B, C). We found that the sex differences in induction time (402.3 ± 37.2 s *vs* 437.7 ± 41.3 s, *P* = 0.2310, *n* = 11 per group; Fig. 2B) and emergence time (199.4 ± 30.8 s *vs* 208.3 ± 37.1 s, *P* >0.9999, *n* = 11 per group; Fig. 2C) were abolished in the MPP group, and the primary change occurred in the males rather than in the females. When ERα was inhibited, males took longer to be anesthetized (341.3 ± 28.6 s, *n* = 8 *vs* 402.3 ± 37.2 s, *n* = 11, *P* = 0.0104; Fig. 2B) and a shorter time to wake up from anesthesia (248.3 ± 37.6 s *vs* 199.4 ± 30.8 s, *P* = 0.0338, *n* = 9 per group; Fig. 2C), while females did not show much change (induction time: 449.1 ± 44.9 s, *n* = 8 *vs* 437.7 ± 41.3 s, *n* = 11, *P* > 0.9999; emergence time: 196.7 ± 33.9 s *vs* 208.3 ± 37.1 s, *P* > 0.9999, *n* = 9 per group; Fig. 2B, C).

To obtain a comparable BSR among male and female mice during maintained deep anesthesia, the concentration of sevoflurane was individually adjusted to 2.4%–2.6% to keep the BSR stable at ~70%. We compared the burst suppression patterns of EEG 15 min after the administration of MPP (Fig. 2D). Representative changes in the EEG spectra are shown in Fig. 2F–I. A significant decrease in burst suppression pattern occurred only in male mice after MPP microinjection. Statistical analysis of the BSR for 5 min before and 15 min after MPP administration confirmed that the BSR in males significantly declined ~7 min after MPP treatment and lasted for at least 8 min (*F*_57, 323_ = 6.426, *P* < 0.0001; Fig. 2E), while that in females did not change significantly. These results suggest a sex-specific regulation of ERα in the MPA under sevoflurane anesthesia. ERα is likely to be involved in the sevoflurane sensitivity in men rather than in women.

Given the involvement of MPA in regulating body temperature [34, 35, 48], and temperature being an important factor for sensitivity to anesthesia, the changes in body surface temperature of male and female mice after MPP injection were measured. As shown in Fig. S1, inhibition of the ERα signal in the MPA significantly increased body surface temperature in males and females (*F*_6, 60_ = 40.28, *P* < 0.0001, *n* = 6 per group), and it took 10–20 min to reach the peak temperature, after which it declined gradually.

### MPP Reduces Spikes in Glutamatergic and GABAergic Neurons in the MPA in Males but not in Females

**Fig. 3:**
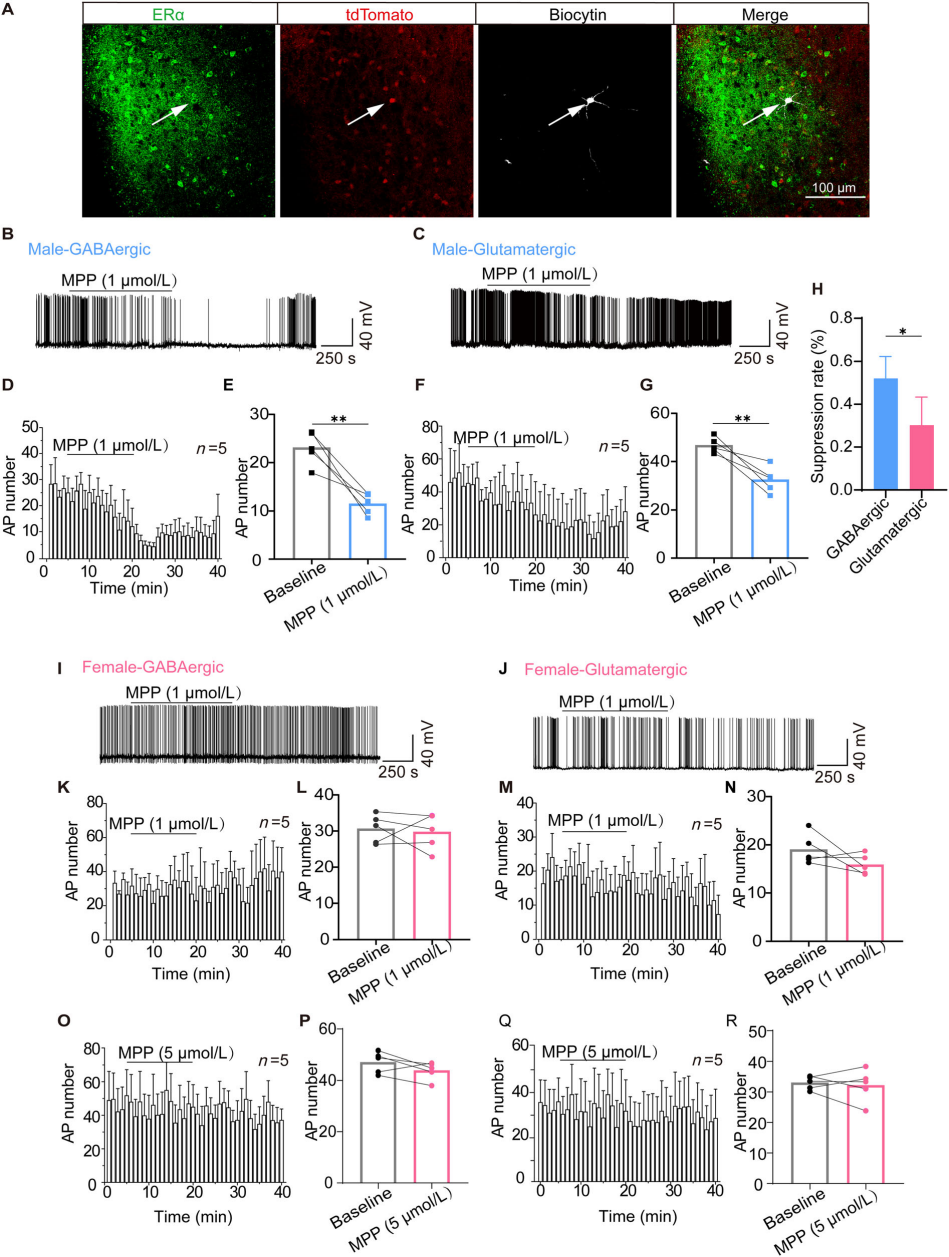
MPP decreases the firing activity of MPA neurons in male but not in female mice. **A** Immunofluorescence test for the co-expression of ERα (scale bar, 100 μm). **B, C, I, J** Representative firing activity over 5 min before, 15 min perfusion, and 20 min wash with MPP (1 μmol/L) application of GABAergic neurons in male (**B**) and female (**I**) mice, and of glutamatergic neurons in male (**C**) and female (**J**) mice. **D, F** Time course of AP number averaged from GABAergic (**D**) and glutamatergic (**F**) neurons in male mice with MPP (1 μmol/L) perfusion. **K, M, O, Q** Time course of AP number averaged from GABAergic (**K, O**) and glutamatergic (**M, Q**) neurons in female mice with MPP (1 μmol/L) and MPP (5 μmol/L) perfusion. **E, G, L, N, P, R** Averaged spike number before 5 min and after 10–15 min of MPP perfusion. Using two-tailed paired Student’s *t*-test: *t*_4_ = 5.347, *P* = 0.0059 for GABAergic neurons in males with MPP (1 μmol/L) (**E**); *t*_4_ = 4.757, *P* = 0.0089 for glutamatergic neurons in males with MPP (1 μmol/L) (**G**); *t*_4_ = 0.3451, *P* = 0.7474 for GABAergic neurons in females with MPP (1 μmol/L) (**L**); *t*_4_ = 1.618, *P* = 0.1809 for glutamatergic neurons in females with MPP (1 μmol/L) (**N**); *t*_4_ = 1.735, *P* = 0.1577 for GABAergic neurons in females with MPP (5 μmol/L) (**P**); *t*_4_ = 0.4295, *P* = 0.6897 for glutamatergic neurons in females with MPP (5 μmol/L) (**R**). **H** Suppression rate of GABAergic and glutamatergic neurons of male mice after injection of MPP (*t*_8_ =2.914, *P* = 0.0195; two-tailed unpaired Student’s *t*-test). AP, action potential.

GABAergic and glutamatergic neurons are the major neuronal subsets in the MPA, both of which have been suggested to play a critical role in modulating arousal behaviors. To examine the specific neuronal effect of ERα in the MPA, whole-cell patch-clamp recordings were performed on the MPA GABAergic and glutamatergic neurons with MPP interventions in acute brain slices from both male and female mice. We identified GABAergic and glutamatergic neurons based on bright tdTomato-fluorescence before patching and checked the co-expression of ERα after recording (Fig. 3A). In male mice, bath application of MPP (1 μmol/L) significantly decreased the number of spike in GABAergic neurons (23.1 ± 3.5 *vs* 11.5 ± 2.2, *P* = 0.0059, *n* = 5 per group; Fig. 3B, D, E) and also slightly reduced the firing rate of glutamatergic neurons (46.8 ± 3.2 *vs* 32.4 ± 5.3, *P* = 0.0089, *n* = 5 per group; Fig. 3C, F, G). Notably, GABAergic neurons fired less by 52 ± 10%, while the spike number of glutamatergic neurons decreased by 30 ± 13% (*P* = 0.0195; Fig. 3H). However, after application of MPP (1 μmol/L) in the slices from females, there was no significant change in the firing rate of the GABAergic neurons (30.6 ± 4.0 *vs* 29.7 ± 5.0, *P* = 0.7474, *n* = 5 per group; Fig. 3I, K, L) or glutamatergic neurons (19.0 ± 3.2 *vs* 15.9 ± 2.1, *n* = 5, *P* = 0.1809; Fig. 3J, M, N). We further increased the MPP concentration to 5 μmol/L, but still did not find a change in the firing activity of GABAergic (47.1 ± 4.2 *vs* 43.9 ± 3.5, *P* = 0.1577, *n* = 5 per group; Fig. 3O, P) or glutamatergic neurons (33.0 ± 2.2 *vs* 32.2 ± 5.4, *P* = 0.6897, *n* = 5 per group; Fig. 3Q, R) in females. These results provide evidence for sex differences in the responses of MPA neurons to ERα inhibition. MPA GABAergic neurons in males exhibited a more profound suppression than glutamatergic neurons.

### ERα Knockdown in GABAergic Neurons of the Male MPA is Sufficient to Eliminate the Sex Difference During Sevoflurane Anesthesia

**Fig. 4:**
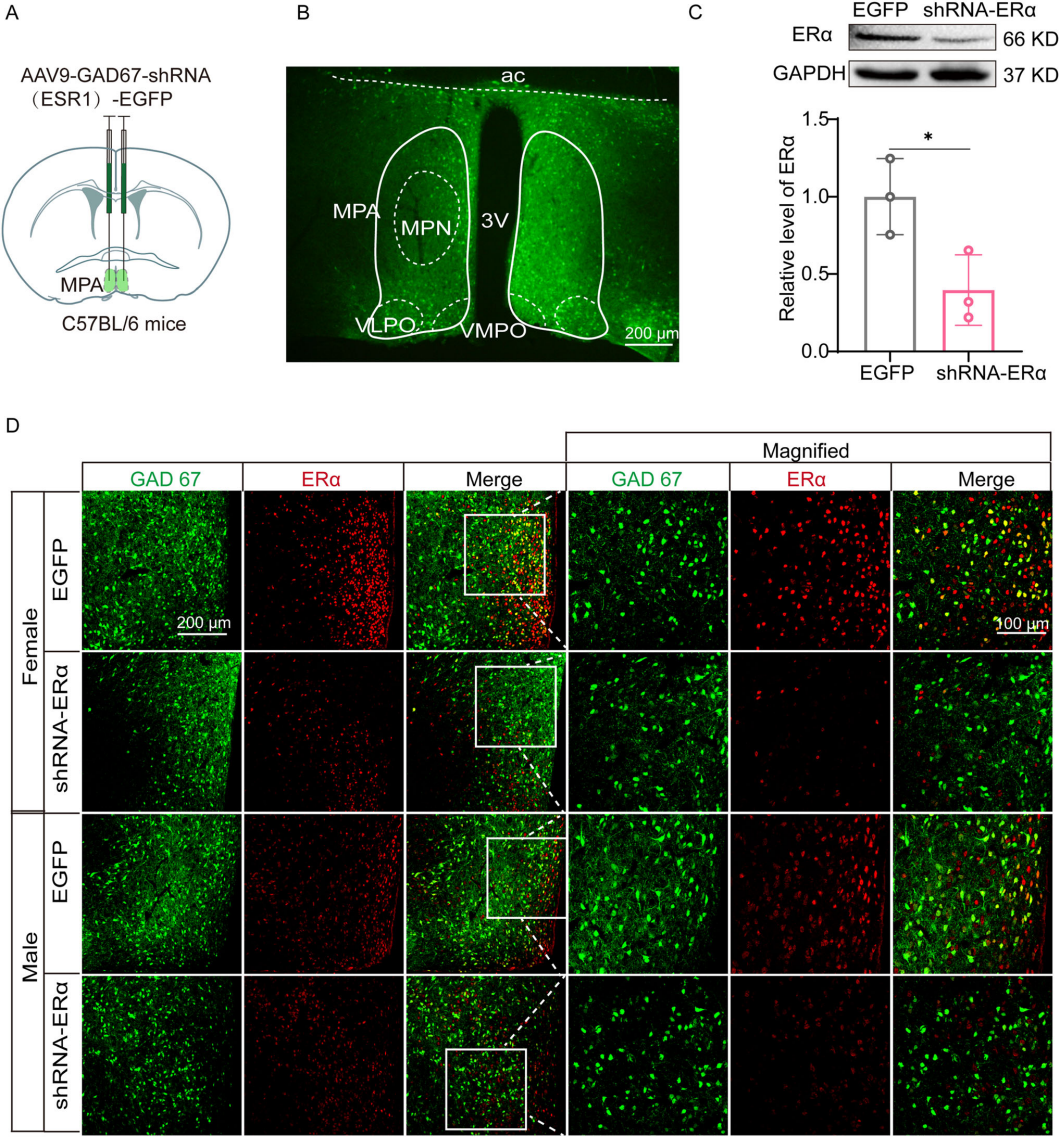
Validation of knockdown efficiency of ERα in the MPA. **A** Schematic of intra-MPA injection in C57BL/6 mice. **B** Expression of the virus (EGFP) in GAD67^+^ neurons in the MPA. **C, D** Western blots (**C**) and immunofluorescence (**D**) showing efficient ERα knockdown in the MPA (*n* = 3; scale bar, 200 μm; *t*_4_ = 3.117, *P* = 0.0356; two-tailed unpaired Student’s *t*-test). 3V third ventricle; ac, anterior commissure; MPA, medial preoptic area; MPN, medial preoptic nucleus; VLPO, ventrolateral preoptic nucleus; VMPO, ventromedial preoptic nucleus.

Inhibitory GABAergic neurons in the MPA are well known for their sleep-promoting nature while excitatory glutamatergic neurons promote wakefulness. Considering that MPP administration in the MPA area produced a stronger suppression in GABAergic neurons and an arousal-facilitation effect on behaviors in male mice, we speculated that ERα in the MPA GABAergic neurons of males could principally mediate the sex difference during sevoflurane anesthesia. To test this hypothesis, we generated and microinjected into the MPA recombinant AAV2/9 expressing a short-hairpin RNA targeting ERα (AAV-GAD67-shERα) driven by the GAD67 promoter (Fig. 4A, B). After a period of 3 weeks for virus expression, double immunofluorescence and western blotting showed a prominent efficiency of ERα knockdown in GABAergic neurons of the MPA (Fig. 4C, D). We also examined the neuronal activity in acute brain slices, and found that the ERα knockdown in the MPA GABAergic neurons eliminated the inhibitory effect of MPP on GABAergic neurons in males (Fig. S2).

**Fig. 5:**
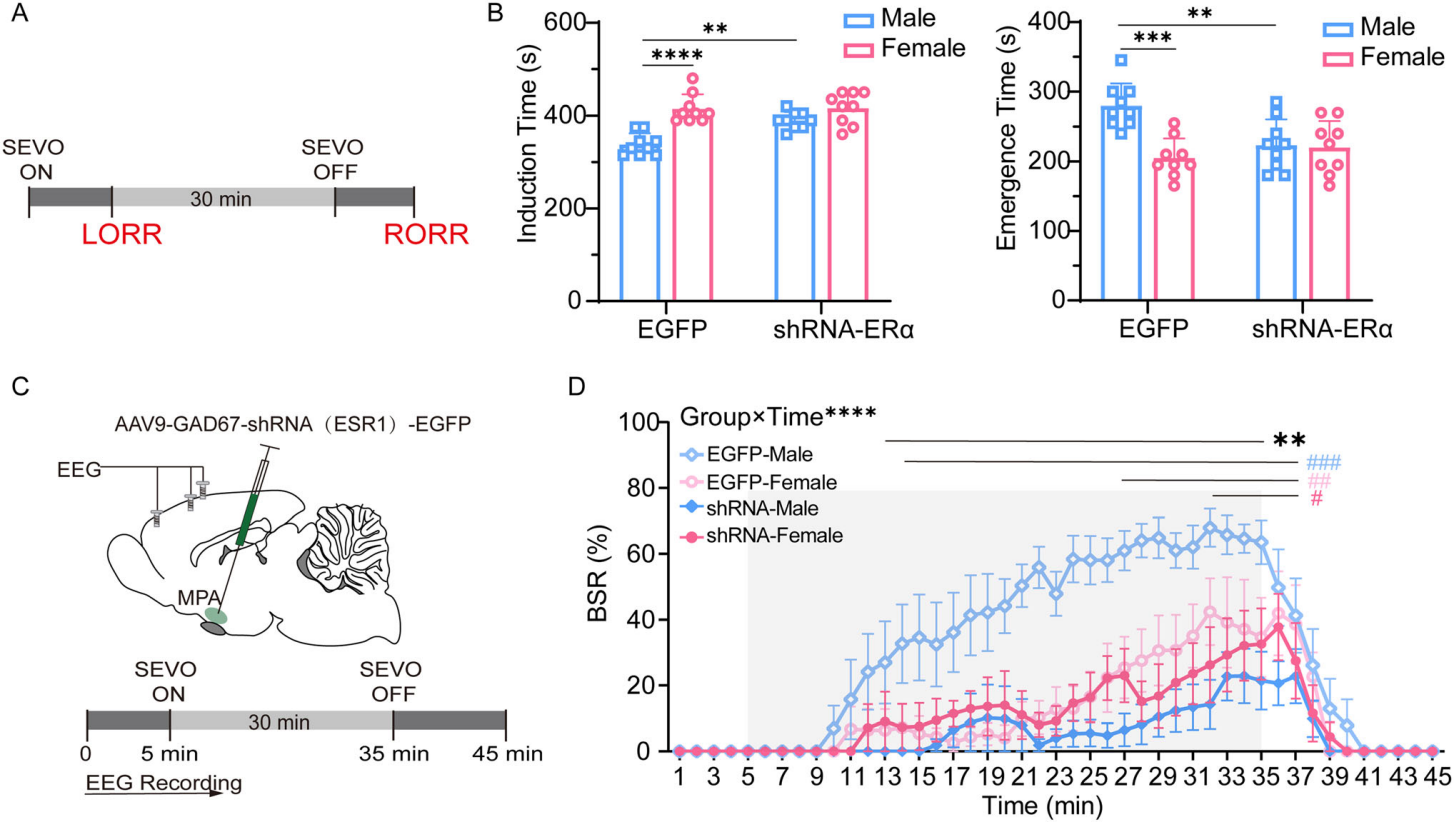
ERα knockdown in MPA GABAergic neurons eliminates the significant gender difference of sevoflurane anesthesia in mice. **A** Protocol for measurement of induction and emergence times. **B** Induction (left) and emergence (right) times in male and female mice in the EGFP and shRNA-ERα group. Using two-way ANOVA followed by *post hoc* Bonferroni’s multiple comparisons: *F*_1,32_ = 7.423, *P* = 0.0103; *t*_32_ = 5.908 (EGFP: male *vs* EGFP: female), *P* < 0.0001; *t*_32_ = 3.981 (EGFP: male *vs* shRNA-ERα: male), *P* = 0.0022 for induction time (left); *F*_1,32_ = 9.968, *P* = 0.0035; *t*_32_ = 4.673 (EGFP: male *vs* EGFP: female), *P* = 0.0003; *t*_32_ = 3.530 (EGFP: male *vs* shRNA-ERα: male), *P* = 0.0077 for emergence time (right). **C** Diagram of a coronal brain section showing virus injection into the MPA of a C57BL/6 mouse (upper) and schematic protocol for EEG recording (lower). **D** BSR throughout a 45-min electroencephalogram recording in EGFP-Male, EGFP-Female, shRNA-Male, and shRNA-Female groups. BSR is calculated every one minute. Using two-way ANOVA followed by *post hoc* Bonferroni’s multiple comparisons: *F*_132,880_ = 2.901, *P* < 0.0001. *Significant difference between male-vehicle and male-MPP groups; ^#^significant difference from the initial value. SEVO, sevoflurane; BSR, burst suppression ratio; LORR, loss of righting reflex; RORR, recovery of righting reflex; MPA, medial preoptic area; EEG, electroencephalogram.

Consistent with the results of pharmacological manipulation with MPP, knocking down the ERα in MPA GABAergic neurons eliminated the sex difference in sevoflurane induction time (390.0 ± 18.4 s *vs* 416.7 ± 34.2 s, *P* = 0.2888, *n* = 9 per group; Fig. 5B) and emergence time (223.3 ± 37.1 s *vs* 220.0 ± 38.2 s, *P* >0.9999, *n* = 9 per group; Fig. 5B). Specifically, male mice with GABAergic ERα knockdown in the MPA required more time to be anesthetized (338.3 ± 23.9 s *vs* 390 ± 18.4 s, *P* = 0.0022, *n* = 9 per group; Fig. 5B) and less time to wake up from anesthesia (280.0 ± 31.9 s *vs* 223.3 ± 37.1 s, *P* = 0.0044, *n* = 9 per group; Fig. 5B) than mice expressing scrambled shRNA (EGFP). In females, there was no significant difference in either induction time (415.0 ± 30.9 s *vs* 416.7 ± 34.2 s, *P* >0.9999, *n* = 9 per group; Fig. 5B) or emergence time (205.0 ± 28.1 s *vs* 220.0 ± 38.2 s, *P* >0.9999, *n* = 9 per group; Fig. 5B) between the EGFP and shRNA-ERα groups.

**Fig. 6:**
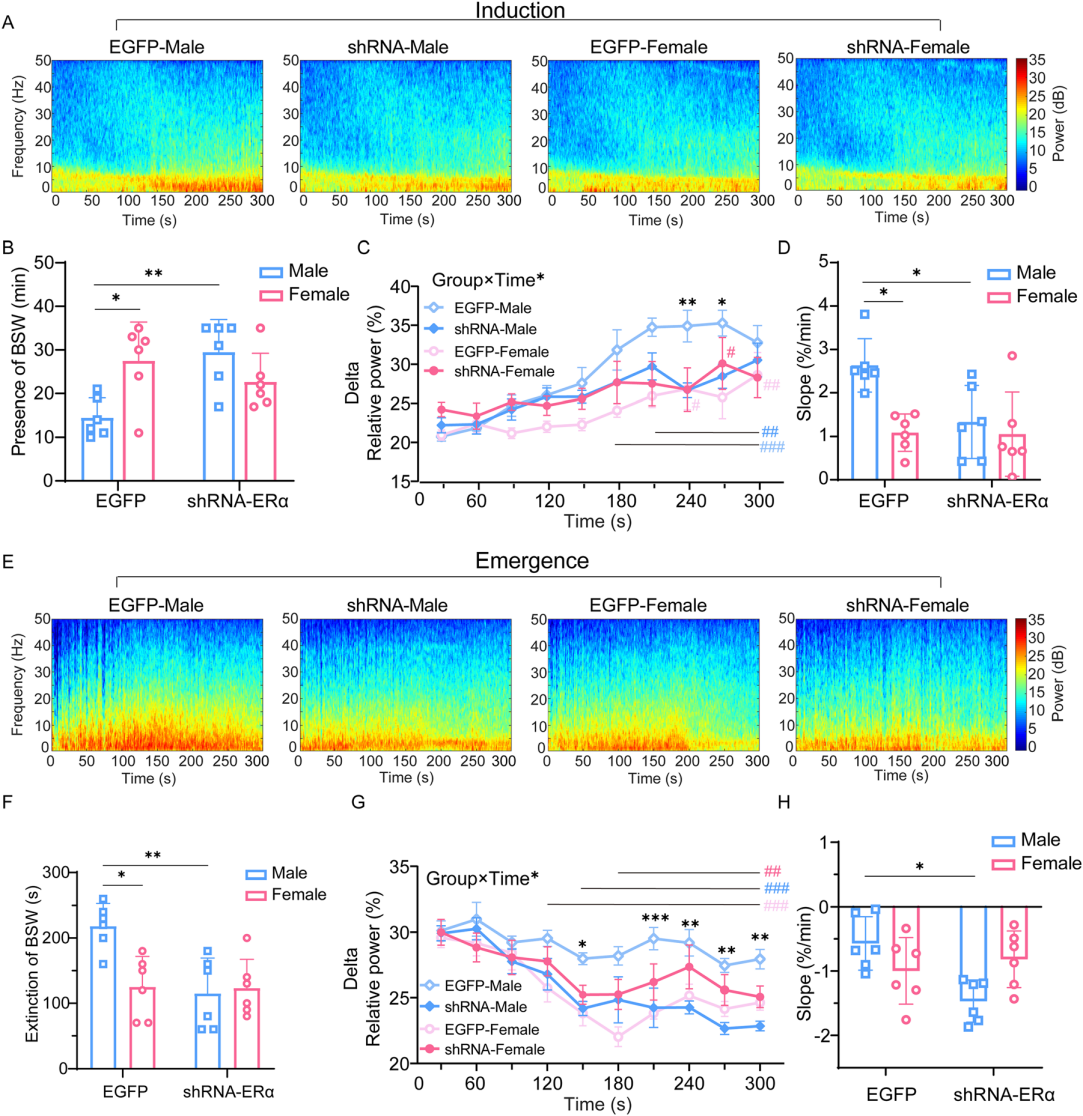
ERα knockdown in MPA GABAergic neurons affects the evolution of EEG signatures during sevoflurane anesthesia in males. **A, E** Averaged spectra in EGFP-Male, EGFP-Female, shRNA-Male, shRNA-Female groups during induction (**A**, 5 min after administration of sevoflurane) and emergence (**E**, 5 min after cessation of administration). **B, F** Presence (**B**) and extinction (**F**) of BSR in male and female mice in the EGFP and shRNA-ERα groups. Using two-way ANOVA followed by *post hoc* Bonferroni’s multiple comparisons: *F*_1,20_ = 11.87, *P* = 0.0026; *t*_20_ = 3.194 (EGFP: male *vs* EGFP: female), *P* = 0.0273; *t*_20_ = 3.686 (EGFP: male *vs* shRNA-ERα: male), *P* = 0.0088 for presence of BSR; *F*_1,20_ = 7.469, *P* = 0.0128; *t*_20_ = 3.548 (EGFP: male *vs* EGFP: female), *P* = 0.0121; *t*_20_ =3.928 (EGFP: male *vs* shRNA-ERα: male), *P* = 0.0050 for extinction of BSR. **C, G** Spectral analysis of relative power in the delta band in EGFP-Male, EGFP-Female, shRNA-Male, shRNA-Female groups (*n* = 6) during induction (**C**, 5 min after administration of sevoflurane) and emergence (**G**, 5 min after cessation of administration). The relative delta power is computed every 30 s. Using two-way ANOVA followed by *post hoc* Bonferroni’s multiple comparisons: *F*_27,180_ =1.750, *P* = 0.0171 for induction (**C**); *F*_27,180_ =1.550, *P* = 0.0496 for emergence (**G**). **D, H** Relative power transition rate in the delta band in EGFP-Male, EGFP-Female, shRNA-Male, shRNA-Female groups (*n* = 6) during induction (**D**) and emergence (**H**). Using two-way ANOVA followed by *post hoc* Bonferroni’s multiple comparisons: *F*_1,20_ = 4.335, *P* = 0.0504; *t*_20_ = 3.600 (EGFP: male *vs* EGFP: female), *P* = 0.0107; *t*_20_ = 3.033 (EGFP: male *vs* shRNA-ERα: male), *P* = 0.0395 for induction (**D**); *F*_1,20_ = 9.373, *P* = 0.0062; *t*_20_ = 3.606 (EGFP: male *vs* shRNA-ERα: male), *P* = 0.0106 for emergence (**H**). *Significant difference between male-vehicle and male-MPP groups; ^#^significant difference from initial value. BSW, burst suppression wave.

Furthermore, MPA GABAergic ERα knockdown significantly decreased the BSR during the maintenance period in male mice, but not in females, which resulted in the disappearance of sex differences in anesthesia depth (*F*_132, 880_ = 2.901, *P* < 0.0001, *n* = 6 per group; Fig. 5D). Compared with the EGFP group, MPA GABAergic ERα knockdown also postponed the presence of a burst suppression pattern in the EEG (14.5 ± 4.5 min *vs* 29.5 ± 7.5 min, *P* = 0.0088, *n* = 6 per group; Fig. 6B) and accelerated the extinction of the pattern (230.8 ± 40.1 s *vs* 115.0 ± 54.3 s, *P* = 0.0050, *n* = 6 per group; Fig. 6F) in males, while females with MPA GABAergic ERα knockdown showed no significant changes in the presence (27.5 ± 8.9 min *vs* 22.77 ± 6.6 min, *P* > 0.9999, *n* = 6 per group; Fig. 6B) or extinction (EGFP: 146.7 ± 65.3 s *vs* shRNA-ERα: 123.3 ± 44.1 s, *P* > 0.9999, *n* = 6 per group; Fig. 6F) of the pattern. As shown in Fig. 6A, with the onset of sevoflurane inhalation, delta waves gradually increased in the EGFP groups in a sex-specific manner. However, MPA GABAergic ERα knockdown largely eliminated this sex difference. We measured the relative power of the delta band every 30 s during the induction stage for 5 min. The male ERα knockdown group showed a decline in both delta power (Fig. 6C) and the rate of increase of delta power (2.6 ± 0.6%/min *vs* 1.3 ± 0.8%/min, *P* = 0.0395, *n* = 6 per group; Fig. 6D) in males, while there was no such effect in females (1.1 ± 0.4%/min *vs* 1.1 ± 0.9%/min, *P* > 0.9999, *n* = 6 per group; Fig. C, D). During the emergence stage, delta oscillation gradually weakened in the EGFP groups in a sex-dependent manner (Fig. 6E). This sex dependence was abolished in the ERα knockdown groups. ERα knockdown reduced the delta power at most time points (Fig. 6G) and accelerated the rate of weakening of the delta wave in males (−0.6 ± 0.4%/min *vs* −1.5 ± 0.3%/min, *P* = 0.0106, *n* = 6 per group; Fig. 6H), while the ERα knockdown groups showed no effect on the delta power dynamics in females (EGFP: −1.0 ± 0.5%/min *vs* shRNA-ERα: −0.8 ± 0.4%/min, *P* > 0.9999, *n* = 6 per group; Fig. 6G, H). Combined with the pharmacological results, MPA GABAergic ERα regulated the sensitivity to sevoflurane anesthesia in males more than females, which could make a pivotal contribution to the differences in behaviors and EEG patterns between male and female mice during general anesthesia.

## Discussion

In the present study, we found sex differences in the behavioral responses and EEG signatures in sevoflurane anesthesia in mice. In particular, males were more prone to lose consciousness during anesthesia induction and maintain a deeper anesthesia and were harder to wake up than females at the same concentration of sevoflurane. Pharmacological inhibition of ERα in the MPA extended the induction time and facilitated the emergence from sevoflurane in males but did not have such effects in females, which consequently abolished the sex bias in sevoflurane anesthesia. *In vitro* electrophysiological tests revealed that the firing activity of GABAergic and glutamatergic neurons of the MPA was inhibited in males but not in females after bath application of an ERα inhibitor, and GABAergic neurons showed greater suppression than glutamatergic neurons. Selective downregulation of ERα in GABAergic neurons of the MPA was sufficient to eliminate the sex difference during sevoflurane anesthesia.

In the last few decades, female rodents have often been neglected in preclinical neuroscience research. A groundbreaking evaluation of biomedical research even reported that male bias was most prominent in terms of neural regulation [49]. The exclusion of females from anesthesia studies also hinders the anesthesia management of female patients because of a lack of knowledge about sex-specific mechanisms. Although a few studies have confirmed that sex plays a role in determining anesthetic requirements and emergence, they have reported conflicting results [5–13]. In our study, we systemically recorded the behavioral and EEG differences during single sevoflurane anesthesia in mice, and found that males were more sensitive to sevoflurane anesthesia and entered deeper anesthesia more easily. Our results are consistent with those of most clinical studies, in which women were found to require a higher propofol infusion rate to maintain general anesthesia [5], a shorter time to emerge from general anesthesia administered with propofol/alfentanil/nitrous oxide complex [6] or inhalational anesthetic [8], and a higher concentration of inhalational anesthesia to maintain BIS levels equivalent to those of men [13]. However, a few basic studies have reported that male rats require larger doses of anesthetics to produce general anesthesia [10, 11], and emerge faster after a single intraperitoneal injection of propofol than female rats [12]. The discrepancy between these studies and our findings could be attributed to the differences in anesthetics, species, and drug delivery strategies [10–12].

Estrogen has been believed to be the leading cause of sex differences in general anesthesia effects for a long time [10]. However, the neuronal targets of this hormone are unclear. The MPA, one of the most established sexually dimorphic structures and a well-known sleep-generating site, is densely enriched in ERα. By microinjection of the selective ERα antagonist MPP into the MPA, we found that the sex difference in sevoflurane anesthesia was completely abolished, which provided evidence for the key role of MPA ERα in the sex difference of sevoflurane anesthesia. Surprisingly, MPP only changed the anesthesia behaviors and EEG signals in males but not in females. Of note, sex differences in the response to manipulation of estradiol signaling are not unprecedented. Indeed, estrogen and its receptors have been traditionally considered to contribute greatly to the unique characteristics of females that are different from those in males, such as reward [50, 51], pain [52, 53], skeletal metabolism [53], emotion-related behaviors [54], and stroke [55]. Vandegrift *et al*. [51] reported that knockdown of ERα in the VTA reduces binge-like ethanol drinking in female mice, but not in males. Indeed, we also expected to find an ERα-mediated anesthesia difference in female mice. However, our findings suggested a regulatory contribution of MPA ERα to the higher sensitivity in male, but not female mice to sevoflurane anesthesia. In the present study, a higher estimated estrogen level in female mice may not fully explain the different responses to MPP between females and males. The differential effects of MPP in female and male mice during sevoflurane anesthesia could be largely attributed to the distinct nature and regulatory mechanism of MPA ERα in the sexes. Although many studies have revealed the various regulatory effects of ERα in males under both physiological and pathological conditions, such as sexual [56] and aggressive behavior [57], pain [43], and temperature regulation [34], only a few studies have compared the roles of ERα or other estrogen receptors between males and females.

In the 1970s, several studies reported that estradiol alters neuronal activity in the preoptic area of the hypothalamus within seconds to minutes [58–61]. Satta *et al*. [50] recently reported that acute treatment with an ERβ agonist induces the expression of the immediate early gene *c-fos* in the nucleus accumbens. In our study, we inhibited the MPA ERα by bath application of MPP in the acute brain slice, and found a significant depression of neuronal activity of GABAergic neurons and glutamatergic neurons of the MPA in male mice, suggesting that ERα activation mediates the excitation of these two types of neurons. However, no changes in firing rate were recorded in the MPA neurons of female mice. In consideration of the possible need for higher MPP by female ERα, we increased the MPP concentration to 5 μmol/L but still found no significant change in the MPA neuronal activity of female mice. A sex-difference of ERα in regulating inhibitory neurotransmission has been reported in the hippocampus. ERα activates postsynaptic mGluR1 to facilitate endocannabinoid release, which then inhibits presynaptic GABAergic terminals [62]. The functional coupling of ERs with metabotropic glutamate receptors (mGluRs) seems to occur only in females, although ER–mGluR complexes exist in both sexes [63]. These reports may not be identical to our findings but provide some hints on the underlying mechanism. With regard to excitatory neurotransmission, the subtypes of ER that mediate hippocampal neural excitation differ between males and females, and presynaptic glutamatergic neurotransmission is regulated by ERβ in females but ERα in males [64], which provides a plausible explanation for the weaker response of glutamatergic neurons to MPP. In addition, GABAergic neurons directly inhibit glutamatergic neurons in the MPA; thus, the greater the suppression of GABAergic neurons by MPP, the greater the disinhibition of glutamatergic neurons [65]. Therefore, the overall effect of MPP may be due to the combination of the direct effect of MPP on glutamatergic neurons and feedforward disinhibition from GABAergic neurons. The classical mechanism of estrogen action involves the slow regulation of gene transcription by acting as a transcription factor [66]. However, more recent studies [67] have confirmed many non-classical estrogen signaling mechanisms that are initiated at the cell membrane and regulate rapid non-genomic action. Estrogen receptors physically interact with mGluRs [68–70], receptor tyrosine kinases [71–73], G proteins [74], and kinases [75] at the cell membrane, forming a functional signaling complex, consequently triggering numerous intracellular signaling cascades, such as gene expression, local protein synthesis, actin polymerization, ion channel dynamics, synaptic physiology, and dendritic spine morphology [76–80]. In our study, the effects of MPP recorded *in vivo* and *in vitro* probably resulted from the rapid non-classical estrogen signaling pathways.

It has been reported that selectively activating estrogen-sensitive MPA ERα^+^ neurons is sufficient to induce a coordinated depression of metabolic rate and body temperature [34]. Body temperature can affect the MAC of inhaled anesthetics, and hypothermia decreases the isoflurane requirement [81]. In our study, inhibition of ERα signaling by microinjection of MPP increased the body surface temperature not only in males but also in females, suggesting that the ERα-mediated body temperature increase may not be a contributor to the sex difference in sevoflurane anesthesia. Interestingly, because the female mice did not show an MPA neuronal response to MPP, the underlying relationship between MPA ERα and temperature elevation in females requires further discussion. First, MPA neurons that regulate anesthesia may not completely overlap with those regulating body temperature. We only recorded the APs in a small population of glutamatergic and GABAergic neurons co-expressing ERα. More importantly, we only recorded APs in neurons that were confined to the MPA, but did not observe that in ventrolateral preoptic nucleus (VLPO). Because of the diffusion of MPP, the VLPO might be involved in the change of temperature in male and female mice, and previous research has reported that modulation of neurons in the VLPO does not alter anesthetic induction or recovery time, while the body temperature changes strikingly [35].

This study has some limitations. We only found sex differences in mice under sevoflurane anesthesia, while different anesthetics with distinct molecular or neural targets may have different effects in the sexes. Because we did not monitor the estrus cycle of the female mice, we do not know yet how much dispersion the fluctuations of sex hormone levels could have contributed to the results in females. In addition, we only focused on the role of ERα in the MPA, but other subtypes of ER, such as ERβ and G protein-coupled ER (also known as GPR30) may have their own effect on sex differences in general anesthesia. Pharmacokinetic gender disparities are prevalent within the respiratory, renal, and endocrine systems. As we did not assess these differences, we could not determine how much the peripheral pharmacokinetic differences affect the sex differences in response to general anesthesia in this study. All of these limitations need to be addressed in future studies.

In conclusion, our study confirmed sex differences in response to sevoflurane anesthesia and found that male mice were more sensitive to sevoflurane anesthesia than females. The ERα of GABAergic neurons in the MPA plays a regulatory role in the anesthesia sensitivity of male mice, which contributes to the sex difference in sevoflurane anesthesia.

## Supplementary Information

Below is the link to the electronic supplementary material.

Supplementary file1 (PDF 914 kb)
