# Supplementary material for: Estrogen Receptor-A in Medial Preoptic Area Contributes to Sex Difference of Mice in Response to Sevoflurane Anesthesia
Source: Neurosci Bull. 2022 Feb 17;38(7):703–19. doi: 10.1007/s12264-022-00825-w (PMC9276904; doi:10.1007/s12264-022-00825-w)
Supplement: Supplementary file 1 — Supplementary file1 (PDF 914 kb) [file 12264_2022_825_MOESM1_ESM.pdf]

Supplementary Figures and Figure Legends

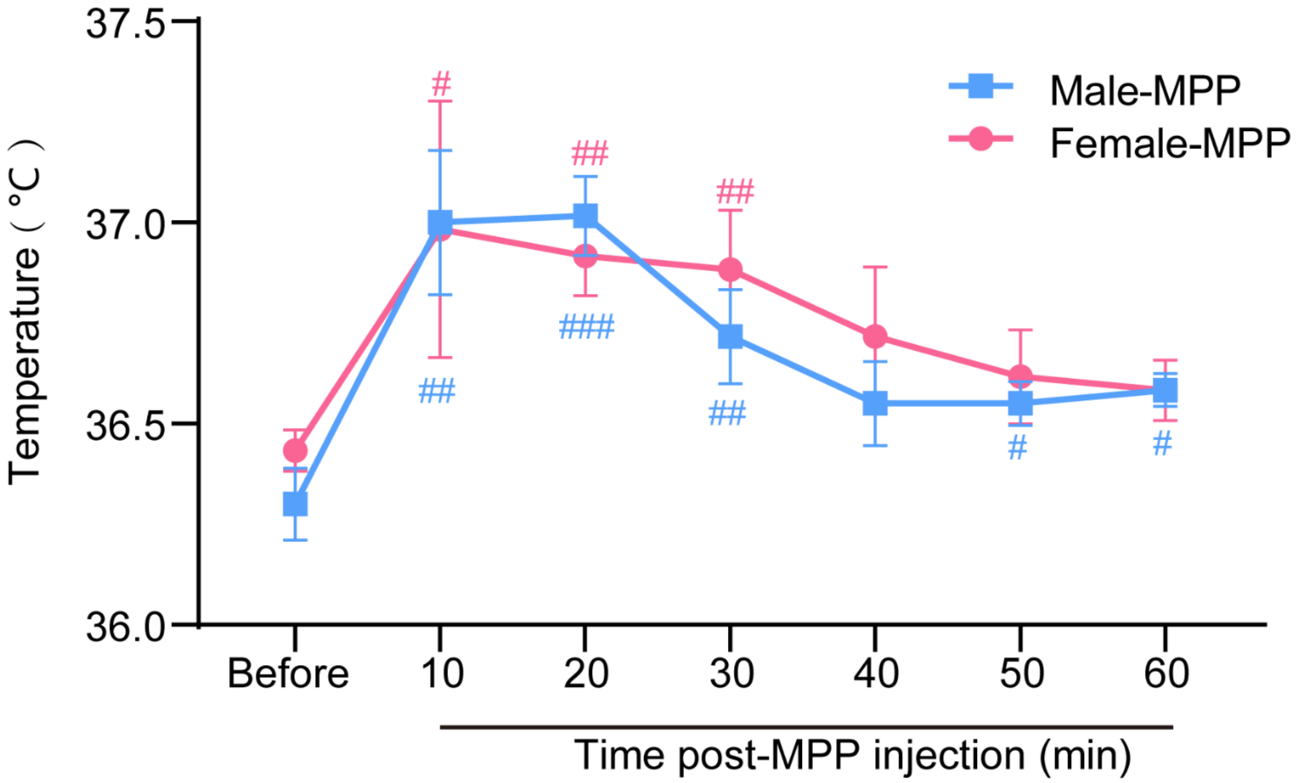

Fig. S1 Inhibition of ERα signaling in the MPA increases body temperature in male and female mice. Body surface temperature in male and female mice before and after injection of MPP (0.3 ng/side). [#]Significant difference in the baseline between male (blue) and female (red) mice using two-way ANOVA followed by *post hoc* Bonferroni's multiple comparisons: $F_{6,60} = 40.28$, $P < 0.0001$, $n = 6$. Data are shown as the mean ± SD.

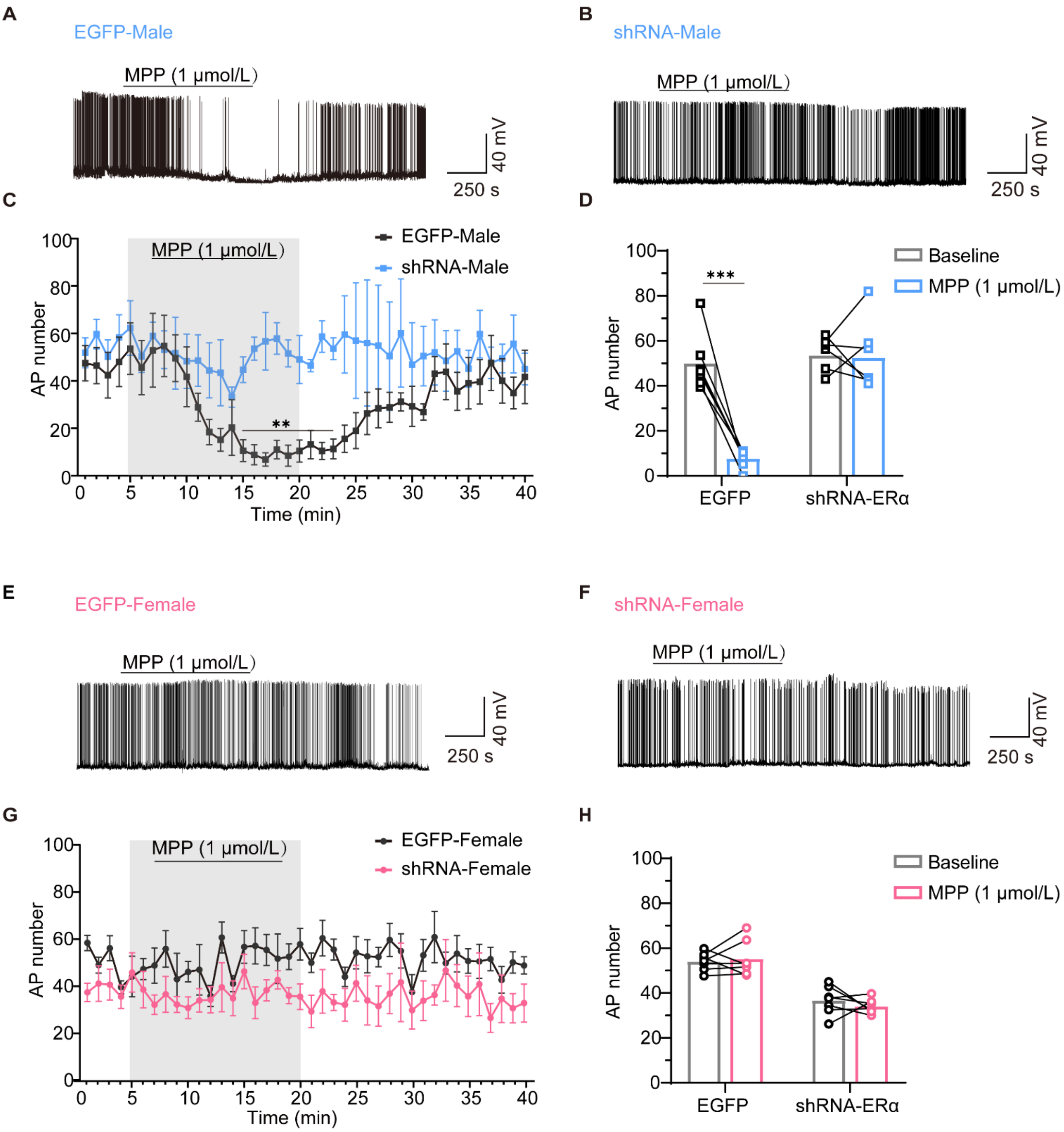

Fig. S2 ERα knockdown in MPA GABAergic neurons eliminates the inhibitory effect of MPP on GABAergic neurons in males. **A, B, E, F** Representative firing activity of GABAergic neurons during 5 min before, 15 min perfusion, and 20 min wash with MPP (1 μmol/L) application in EGFP-Male (**A**), shRNA-Male (**B**), EGFP-Female (**E**), and shRNA-Female groups (**F**). **C** Time course of AP number in GABAergic neurons of the EGFP-Male and shRNA-Male groups over MPP (1 μmol/L)

perfusion. Using two-way analysis of variance (ANOVA) followed by *post hoc* Bonferroni's multiple comparisons: $F_{39,468} = 1.303$, $P = 0.1095$; *significant difference between EGFP-Male and shRNA-Male groups. **D** Averaged spike numbers during 5 min before MPP perfusion and during the last 5min of MPP perfusion in the EGFP-Male and shRNA-Male groups. Using two-way ANOVA followed by *post hoc* Bonferroni's multiple comparisons: $F_{1,12} =23.92$, $P = 0.0004$; $t_6 = 7.472$ [EGFP: Baseline *vs* EGFP: MPP (1 μmol/L)], $P <0.0001$; $t_6 = 0.1821$ [shRNA-ERα: Baseline *vs* shRNA-ERα: MPP (1 μmol/L)], $P >0.9999$. **G** Time course of AP number in GABAergic neurons of the EGFP-Female and shRNA-Female groups over MPP (1 μmol/L) perfusion. Using two-way ANOVA followed by *post hoc* Bonferroni's multiple comparisons: $F_{39,468} = 0.5382$, $P = 0.9904$. **H** Averaged spike numbers during 5 min before MPP perfusion and during the last 5 min of MPP perfusion in the EGFP-Female and shRNA-Female groups. Using two-way ANOVA followed by *post hoc* Bonferroni's multiple comparisons: $F_{1,12} = 0.7172$, $P = 0.4136$; $t_6 = 0.3955$ [EGFP: Baseline *vs* EGFP: MPP (1 μmol/L)], $P >0.9999$; $t_6 = 0.8584$ [shRNA-ERα: Baseline *vs* shRNA-ERα: MPP (1 μmol/L)], $P = 0.7983$. AP, action potential.
